# Gut microbiome-based machine learning model for early colorectal cancer and adenoma screening

**DOI:** 10.1186/s13099-025-00750-z

**Published:** 2025-10-08

**Authors:** Yi-Jian Tsai, Wei-Ni Lyu, Nai-Shun Liao, Pei-Chun Chen, Mong-Hsun Tsai, Eric Y. Chuang

**Affiliations:** 1 Graduate Institute of Biomedical Electronics and Bioinformatics, National Taiwan University, Taipei, Taiwan; 2 Division of Colorectal Surgery, Department of Surgery, Fu Jen Catholic University Hospital, New Taipei City, Taiwan; 3 Institute of Biotechnology, National Taiwan University, Taipei, Taiwan; 4 Department of Mathematics and Information Education, National Taipei University of Education, Taipei, Taiwan

**Keywords:** Colorectal cancer, Adenoma, Gut microbiome, Machine learning, Random forest, Microbial risk score, Non-invasive screening

## Abstract

Colorectal cancer (CRC) is a major source of cancer-related deaths, but early detection at the adenoma stage markedly improves outcomes. Existing tools such as colonoscopy and fecal immunochemical testing (FIT) are invasive or insensitive to early lesions. To develop a non-invasive screening strategy, we analyzed five publicly available 16 S rRNA sequencing datasets from North American and East Asia. Using Analysis of Compositions of Microbiome with Bias Correction (ANCOM-BC) and chi-square testing, we identified 109 discriminatory microbial taxa and trained random forest (RF) classification models to distinguish healthy controls, adenomas, and CRC. The models performed well in internal validation (AUC = 0.90, 95% CI: 0.869–0.931) and external validation (AUC = 0.82), indicating cross-population generalizability. We further developed a microbial risk score (MRS), inspired by polygenic risk score (PRS), methodology, which was significantly elevated in CRC across cohorts. Enrichment of CRC-associated pathogens such as *Fusobacterium nucleatum* and *Porphyromonas gingivalis* supports the biological relevance of the findings. These results demonstrate the potential of gut microbiome signatures combined with machine learning as scalable, non-invasive approach for early CRC and adenomas detection.

**Supplementary Information:**

The online version contains supplementary material available at 10.1186/s13099-025-00750-z.

## Background

Colorectal cancer (CRC), characterized by uncontrolled cell growth in the colon or rectum, is the third most frequently diagnosed cancer globally and the fourth leading cause of cancer-related mortality [1, 2]. CRC typically develops from precancerous lesions such as adenomatous polyps, progressing into advanced adenoma (AA) and invasive cancers if undetected [3]. Early detection, particularly at the adenoma stage, significantly improves prognosis and patient survival [4]. However, existing screening modalities, including colonoscopy and fecal immunochemical tests (FIT) [5, 6], face considerable limitations. Colonoscopy, though effective, is invasive and associated with patient discomfort and poor compliance [7], while FIT suffers from limited sensitivity (approximately 23.3–46%) in detecting AA [8] and generates substantial false-positive results [9]. Therefore, there is an urgent clinical need for more accurate, non-invasive, and acceptable alternatives for CRC screening. Additionally, recent epidemiological studies have provided further insights into CRC patient subgroups, including an analysis of anatomical location, risk factors, and outcomes of lower gastrointestinal bleeding in CRC patients [10], and a population-based study of maternal and perinatal outcomes in pregnant patients with CRC [11].

Emerging evidence has demonstrated the critical involvement of gut microbiome dysbiosis in colorectal carcinogenesis [12], mediated by chronic inflammation [13], altered metabolite production [14], and microbial genotoxins (BTGX) leading to DNA damage [15]. Microbial taxa such as *Fusobacterium nucleatum* and *Porphyromonas gingivalis* are consistently associated with CRC development [16], suggesting their potential as biomarkers for non-invasive CRC and adenoma detection. Recent advancements in 16 S rRNA sequencing technologies have facilitated deeper insights into microbiome composition and functions, offering promising avenues for developing microbiome-based biomarkers for early CRC risk stratification [17, 18].

In addition to diagnostic applications, recent studies have explored the broader relevance of gut microbiota in CRC pathogenesis and host regulation. For instance, Qingjie Fuzheng Granules (QFG) were shown to inhibit colitis-associated CRC progression in mice through modulation of the NOD2/nuclear factor kappa-B (NF-κB) pathway and rebalancing of gut microbial communities [19]. Separately, oxaliplatin resistance in CRC has been linked to NAD⁺ depletion and SIRT1 downregulation [20], which promotes glycolytic reprogramming. Importantly, gut microbiome can modulate SIRT1 activity—such as via metabolites like hydrogen sulfide (H₂S) and microbial-derived compounds that influence sirtuin signaling in host tissue [21, 22]—suggesting a plausible link between microbiota-mediated metabolism and glycolytic rewiring during chemotherapy resistance. Beyond oncology, dietary supplementation with *Litsea cubeba* essential oil in pigs has been shown to enhance antioxidant capacity and nutrient absorption via alterations in gut microbiota [23]. These studies highlight the biological and physiological significance of microbiome-host interactions in both disease and health.

Despite these advances, translating microbiome data into clinical screening tools remains challenging due to the microbiome’s complexity and variability influenced by diet, environment, and host genetics [17]. To overcome these barriers, this study integrates microbiome data with advanced machine learning (ML) techniques to develop a novel screening pipeline capable of accurately detecting early-stage CRC and colorectal adenomas. Specifically, we employed random forest (RF) classifiers to construct predictive models and applied robust statistical methods such as Analysis of Compositions of Microbiomes with Bias Correction (ANCOM-BC) to minimize false positives and identify reliable microbial biomarkers. Furthermore, we introduced a microbial risk score (MRS) [24], inspired by polygenic risk scores (PRS), to quantify CRC risk based on microbiome profiles. This innovative approach aims to substantially enhance non-invasive CRC screening, improve early diagnosis rates, and ultimately patient outcomes.

## Methods

### Published dataset collection

To develop and validate a gut microbiome-based ML model for early CRC and adenoma screening, publicly available stool microbiome datasets containing 16 S rRNA sequencing data from CRC, non-advanced adenomas, AA, and healthy control subjects diagnosed via colonoscopy were collected. A total of five publicly available 16 S rRNA sequencing datasets (Baxter, Dadkhah, Zackular, Yang, and Cong) [25–29], primarily from the USA and Canada, were included and analyzed to minimize geographical bias (Supplementary Table 1).

Three datasets (Baxter, Dadkhah, Zackular) served as primary discovery/validation cohorts for feature selection, RF classifier training, and MRS development. Specifically, the Baxter and Dadkhah datasets were used for RF training and internal 10-fold cross-validation, whereas the Zackular dataset was used exclusively for independent external validation to assess model generalizability.

Two Chinese cohorts (Yang and Cong) were used solely for cross-regional validation of the binary MRS model. While both datasets included adenoma cases, the Yang dataset only provided processed ASV tables without raw sequencing data, preventing standardized reprocessing. This role separation ensured transparency in dataset usage, avoided information leakage between training and testing phases, and enabled the assessment of cross-population transferability.

Participants were included if they had undergone colonoscopy with a confirmed diagnosis of CRC, AA, non-advanced adenoma, or healthy control status, and had available stool 16 S rRNA sequencing data with associated metadata. Exclusion criteria were: prior history of CRC or other gastrointestinal malignancies; inflammatory bowel disease; antibiotic, probiotic, or prebiotic use within three months prior to sample collection; incomplete metadata; and sequencing runs with insufficient read depth (< 10,000 reads after quality control). For all publicly available datasets, these criteria were applied to the extent possible based on the metadata provided. Detailed cohort-specific inclusion/exclusion criteria were not uniformly available from the original studies, and any additional restrictions beyond the core criteria followed those described in the corresponding primary publications.

Data and metadata were obtained from the National Center for Biotechnology Information (NCBI) Sequence Read Archive (SRA) database, and incomplete samples were excluded. The Baxter [26] and Dadkhah [28] datasets were used to train RF classifiers, while Zackular dataset [25] was employed for external validation to assess model generalizability. The MRS model was constructed using the Baxter dataset and validated using the Zackular dataset to reduce technical variability.

The Zackular dataset [25] included 90 participants (30 CRC, 30 non-advanced adenoma, 30 controls) recruited in the United States. The mean age (± SD) was 55.3 ± 9.2, 61.3 ± 11.1, and 59.4 ± 11.0 years for the healthy, non-advanced adenoma, and CRC groups, respectively. Across groups, 37–70% were men, and the majority were non-Hispanic White (70–93%). Mean BMI ranged from 26.6 to 30.7 kg/m². Cancer stage information was not reported.

Across the three primary datasets used for model development (Supplementary Table 2), the total sample size was 1,181, comprising 454 controls, 373 non-advanced adenomas, 197 advanced adenomas, and 157 CRC cases. The Baxter and Dadkhah datasets contained more controls than CRC cases, whereas Zackular had balanced class sizes. The Yang dataset was balanced (50 CRC, 50 controls), while Cong was slightly imbalanced (10 CRC, 11 controls). Post-hoc power analysis was performed based on the observed effect sizes in alpha diversity and microbial feature abundance between CRC and non-CRC groups. Using G*Power 3.1 and the primary dataset sample sizes (non-CRC *n* = 1,024; CRC *n* = 157), we determined that, under the assumption of a moderate effect size (Cohen’s d = 0.4) and α = 0.05, the study achieved 99.66% power, which is well above the conventional 80% threshold. This is well above the conventional 80% threshold, supporting that our analyses and model development were adequately powered to detect moderate group differences.

### Data preprocessing

Raw 16 S rRNA sequencing data were processed using the Easy Microbiome Analysis Platform (EasyMAP) [30], incorporating the QIIME2 pipeline [31]. Sequencing reads were demultiplexed, trimmed, merged, and denoised using DADA2 to generate high-quality amplicon sequence variants (ASVs). Taxonomic classification of ASVs was performed with pre-trained classifiers based on the SILVA reference database. As the discovery and validation datasets targeted different 16 S rRNA hypervariable regions (V1–V3, V3–V4, or V4), region-specific classifiers were trained and evaluated separately to minimize primer-driven compositional bias. Feature selection was also performed within each region so that only taxa consistently detected with the corresponding primer set were included in model construction.

ASV tables were normalized by total-sum scaling (TSS), and binary appearance data were created by converting ASV presence to 1 (present) or 0 (absent). The appearance ratio was calculated as the percentage of samples in which an ASV was observed within each group. Microbial community composition and inter-group variation were visualized using the phyloseq R package (v1.42.0) within the Bioconductor (v3.17), including phylum-level bar plots and principal component analysis (PCA).

Due to limited AA representation in some datasets—particularly the complete absence of AA cases in certain validation cohorts (e.g., Zackular)—the AA group was combined with the non-advanced adenoma group for feature selection, model development, and MRS calculation. This strategy ensured sufficient statistical power and is biologically justified, as both AA and non-advanced lie along the adenoma–carcinoma sequence and already exhibit detectable dysbiotic shifts at the early-lesion stage. Adenoma-associated microbial markers have been shown to be reproducible across cohorts, and classifier panels trained on mixed adenomas populations perform comparably in distinguishing both advanced and non-advanced lesions [25, 32–34]. Similar grouping approaches have been widely adopted in large-scale cancer microbiome studies to enhance statistical robustness while maintaining biological plausibility.

Taxa present in fewer than 5% of samples were removed prior to analysis to reduce sparsity-driven artifacts, and samples with low read depth were excluded from downstream analyses. Missing metadata were handled by excluding incomplete samples, and no imputation was applied to taxonomic abundances.

### Differential abundance analysis and feature selection

To enhance model performance and reduce dimensionality, differential abundance analysis (DAA) was performed to identify CRC-associated microbial features. ANCOM-BC (Bioconductor v3.17, v2.0.3) [35] was used, which applies the Benjamini–Hochberg method to adjust for multiple comparisons. All p-values from differential abundance testing were adjusted using the Benjamini–Hochberg method to control the false discovery rate (FDR), with FDR < 0.05 considered statistically significant. Given the relatively large number of taxa tested (*n* = 109), we also applied a stricter preliminary filter of raw *p* < 0.01 in both ANCOM-BC and chi-square tests to further ensure robustness and reduce the likelihood of spurious associations. This process yielded 109 candidate biomarkers, which were subsequently used for the development of the RF classifier and MRS model (Supplementary Fig. 1).

### ML model development and validation

Two complementary models were developed: a classification model using RF classifiers and a risk stratification model using the MRS. RF models were constructed using scikit-learn (v1.2.2), leveraging ensemble decision trees to reduce overfitting. A nested, stratified 10-fold cross-validation (CV) framework was used, in which the inner loop performed feature selection, MRS threshold determination, and hyperparameter tuning, and the outer loop provided unbiased performance estimates from held-out folds. Participants were split at the subject level, with class proportions preserved and a fixed random seed to ensure reproducibility. Hyperparameters were optimized via grid search over the number of trees, maximum tree depth, minimum samples per split, feature subset size, and class weight settings. All preprocessing—including differential abundance filtering, normalization, and threshold determination—was performed exclusively on training folds and then applied unchanged to the corresponding test fold to prevent information leakage.

To assess robustness to parameter choices, we conducted a sensitivity analysis by systematically varying key hyperparameters. Across the tested configurations, out-of-fold AUC, sensitivity, and specificity showed only minimal variation, indicating that the RF classifier is stable across a broad range of hyperparameter settings. For datasets lacking an external validation cohort, stratified 10-fold CV was used with the same anti-leakage protocol. In external validation, no resampling or balancing was applied to reflect the real-world prevalence of CRC, adenoma, and controls. Model performance metrics included accuracy, area under the receiver operating characteristic curve (AUC), sensitivity, specificity, calibration (Brier score and slope), and external validation performance. Features selected via ANCOM-BC and chi-square tests were used as model inputs, and class weights were adjusted during training to further compensate for imbalance. External validation was conducted using the independent Zackular dataset. For all metrics derived from 10-fold cross-validation, 95% confidence intervals (CIs) were calculated from fold-wise results using the formula: mean ± 1.96 × (SD/√n), where *n* = 10. This approach provides a transparent quantification of variability and facilitates clearer interpretation of model robustness.

### MRS calculation

The MRS is conceptually analogous to the PRS, which combines multiple genome-wide significant variants into a single quantitative measure of genetic risk. Similarly, the MRS condenses information from a disease-associated microbial sub-community into one interpretable score, capturing the collective characteristics of relevant taxa rather than focusing on individual features [24]. This approach leverages within-sample alpha diversity to reflect the complexity of microbe–microbe and microbe–host interactions, providing a more holistic representation of microbiome-related risk.

In our two-stage framework, candidate ASVs were first identified in the discovery cohort through differential abundance analysis (ANCOM-BC, ALDEx2 [36], MaAsLin2 [37]; FDR < 0.05). We then applied a pruning-and-thresholding (P + T) procedure to select an optimal sub-community: for each p-value cut-off, we calculate Shannon, Simpson, and observed-ASV indices and construct corresponding MRS models. Model definition was determined within a nested CV framework, where an inner loop selected the optimal P + T threshold and alpha-diversity index, and an outer 10-fold CV generated unbiased out-of-fold predictions. All preprocessing and index computation steps were performed only within training folds and applied unchanged to test folds. Receiver-operating characteristic (ROC) curves were generated using cross-validated predictions within the discovery cohort, and the threshold yielding the highest cross-validated AUC was selected. In the validation phase, this threshold was applied unchanged to the independent validation cohort to avoid information leakage. The final MRS was recalculated in both cohorts, and group differences were assessed using unpaired two-tailed Student’s t-tests. This design integrates multiple microbial features into a single robust score while minimizing selection bias and ensuring transferability across populations.

## Results

### Gut microbial composition differentiates control, adenoma, and CRC groups

**Fig. 1:**
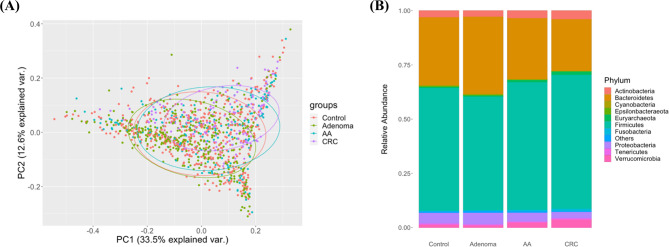
Microbiome composition and diversity across control, adenoma, AA, and CRC groups. (**A**) PCA showing the variance explained by the first two principal components, which accounted for 33.5% and 12.6% of the variance. (**B**) Phylum-level relative abundance of gut microbiome across the four groups. Y-axis: stacked relative abundance of the four groups

We analyzed stool microbiome profiles from control, non-advanced adenoma, AA, and CRC patients from three datasets were included (Supplementary Table 2). PCA reveals clear separations, particularly between CRC and control/adenoma groups (Fig. 1A). At the phylum-level, Firmicutes, Bacteroidetes, Proteobacteria, Actinobacteria, and Verrucomicrobia were predominant across all groups, with compositional differences observed between control, adenoma, and CRC samples (Fig. 1B).

### Identification of microbial biomarkers for CRC and adenoma screening

**Fig. 2:**
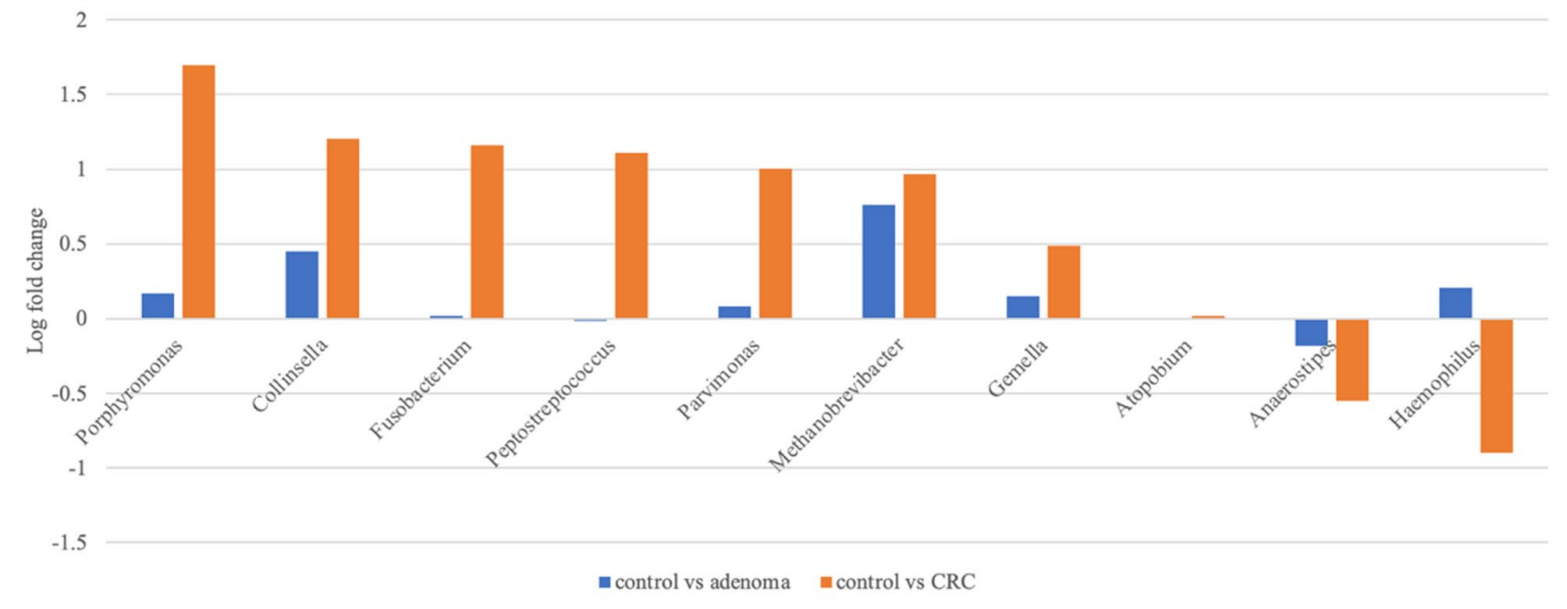
Log fold change of key bacterial genera between control, adenoma, and CRC groups. Y-axis: Log fold change in different comparisons (control vs. adenoma, control vs. CRC). Orange bars represent differential abundance between control and CRC groups, while blue bars show differences between control and adenoma groups. Genera were selected based on statistical significance in both ANCOM-BC and chi-square tests

DAA identified key microbial biomarkers across control, adenoma, and CRC groups. Log fold changes of bacterial genera were calculated, with several taxa showing distinct enrichment in CRC samples. The top-ranked genera included *Porphyromonas*, *Peptostreptococcus*, *Parvimonas*, *Fusobacterium*, *Haemophilus*, *Atopobium*, and *Collinsella*, all of which exhibited significant differences between CRC and control groups (Fig. 2).

**Fig. 3:**
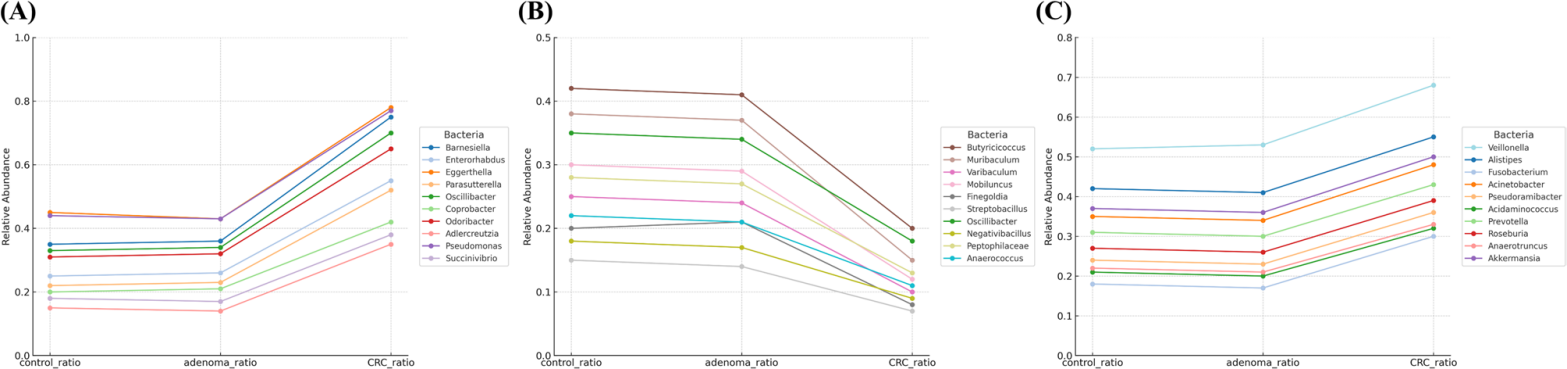
Relative abundance patterns of selected bacterial genera across control, adenoma, and CRC groups. (**A**) Genera with increased abundance in CRC. (**B**) Genera with decreased abundance in CRC. (**C**) Genera with decreased abundance in adenoma. The Y-axis represents the proportion of samples within each group in which the corresponding genus was observed

Relative abundance analysis further revealed genera enriched in CRC samples (Fig. 3). A total of 109 biomarkers were identified after Benjamini–Hochberg FDR adjustment (FDR < 0.05) and additional filtering with raw *p* < 0.01 in both ANCOM-BC and chi-square tests. Among them, the top seven genera—*Porphyromonas*, *Peptostreptococcus*, *Parvimonas*, *Fusobacterium*, *Haemophilus*, *Atopobium*, and *Collinsella*—were selected as representative features and incorporated into downstream ML model development (Supplementary Table 3).

### Performance of microbial ML models in CRC and adenoma screening

To assess model performance, RF classifiers were trained on the Baxter and Dadkhah datasets using selected microbial features, and externally validated with the Zackular dataset. For all metrics derived from 10-fold cross-validation, we also report the corresponding 95% CIs calculated from fold-wise results, allowing clearer interpretation of model robustness. Sensitivity analysis across tested parameter ranges showed consistent performance, with AUC variation < 2%, confirming model robustness. For CRC versus control classification, the model achieved an AUC of 0.90 (95% CI: 0.869–0.931) in 10-fold CV and 0.82 in external validation, with high specificity (0.97 internal, 0.96 external) but reduced sensitivity in external validation. The adenoma versus CRC model also showed strong performance (AUC 0.90, 95% CI: 0.888–0.912 internal; 0.84 external). In contrast, the control versus adenoma classification yielded lower accuracy (AUC 0.63, 95% CI: 0.599–0.661 internal; 0.62 external, Table 1). Combining adenoma and CRC groups versus controls (where the adenoma group included both AA and non-advanced adenoma cases) increased sensitivity (0.87, 95% CI: 0.851–0.889) in internal 10-fold CV and 0.97 in external validation, though with a corresponding decrease in specificity (Table 2). This grouping approach was necessary to achieve adequate statistical power given the absence of AA cases in some external cohorts, and is consistent with prior microbiome CRC studies [25, 32–34].

**Table 1:** Performance metrics of random forest classifiers for pairwise binary classification strategies

Strategy	Validation method	AUC	Accuracy	Sensitivity	Specificity
**Control vs. Adenoma**	**10-fold cross validation**	0.63 (SD=0.05) (0.599, 0.661)	0.59 (SD=0.04) (0.565, 0.615)	0.79 (SD=0.08) (0.740, 0.840)	0.40 (SD=0.09) (0.344, 0.456)
	**External validation**	0.62	0.55	0.73	0.37
**Adenoma vs. CRC**	**10-fold cross validation**	0.90 (SD=0.02) (0.888, 0.912)	0.86 (SD=0.05) (0.829, 0.891)	0.38 (SD=0.12) (0.307, 0.453)	0.97 (SD=0.01) (0.964, 0.976)
	**External validation**	0.84	0.66	0.33	0.98
**Control vs. CRC**	**10-fold cross validation**	0.9 (0.869, 0.931)	0.85 (0.825, 0.875)	0.43 (0.348, 0.512)	0.97 (0.957, 0.983)
		(SD=0.05)	(SD=0.04)	(SD=0.13)	(SD=0.02)
	**External validation**	0.82	0.67	0.38	0.96

SD: standard deviation

Bold text is used only to highlight the classification strategies and performance metrics of primary interest

**Table 2:** Performance metrics of the random forest classifier for the control vs. adenoma + CRC binary classification strategy

Strategy	Validation method	AUC	Accuracy	Sensitivity	Specificity
**Control vs. Adenoma + CRC**	**10-fold cross validation**	0.71 (0.679, 0.741)	0.66 (0.641, 0.679)	0.87 (0.851, 0.889)	0.36 (0.323, 0.397)
		(SD=0.05)	(SD=0.03)	(SD=0.03)	(SD=0.06)
	**External validation**	0.8	0.72	0.97	0.21

SD: standard deviation

Boldface is used only to highlight the classification strategies and performance metrics of primary interest

### CRC risk stratification using MRS

**Fig. 4:**
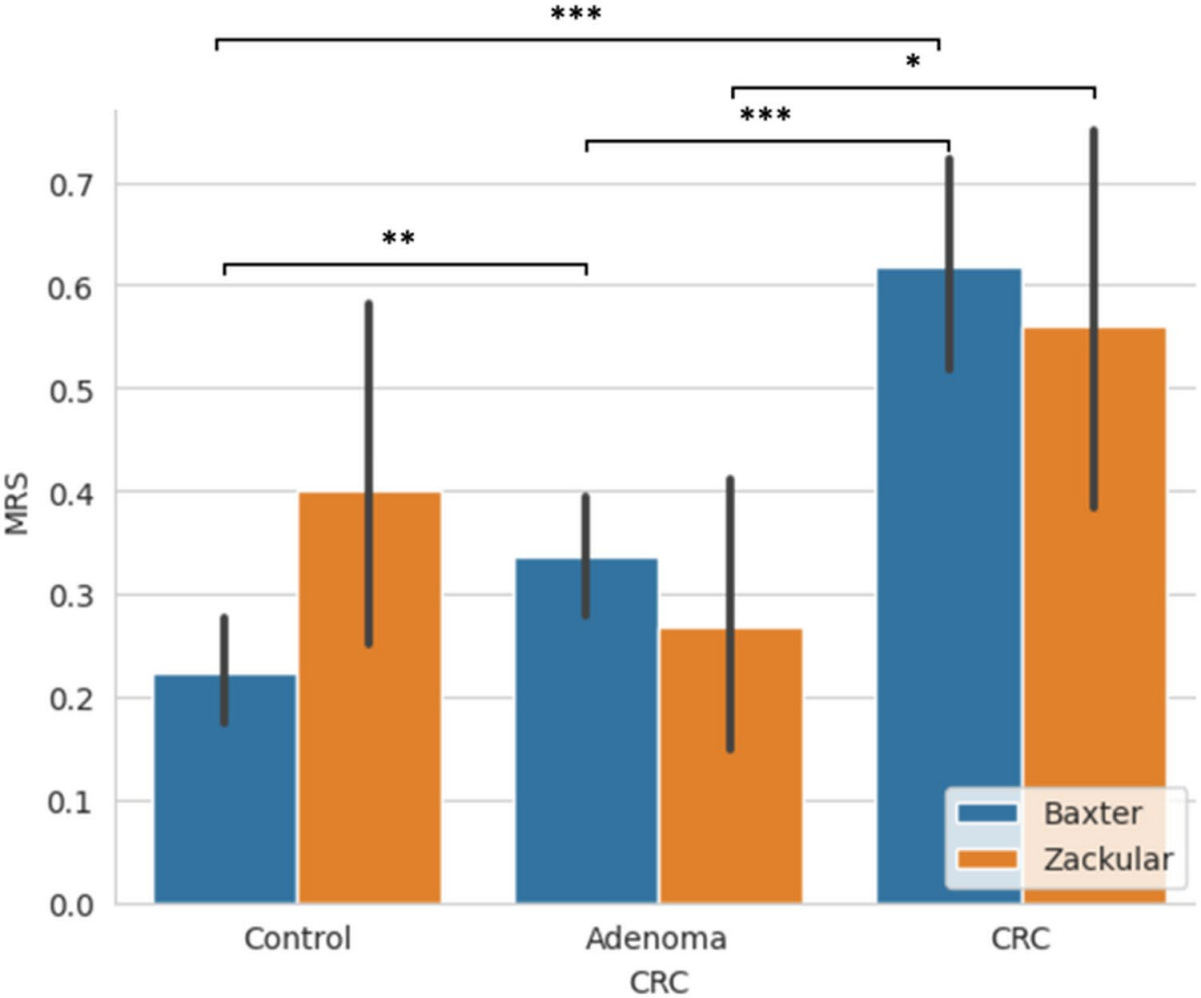
MRS across control, adenoma, and CRC Groups in discovery and validation cohorts. Y-axis: MRS score; X-axis: Three sample groups across two datasets; Error bars: 95% Confidence Interval (CI); *p ≤ 0.05, **p ≤ 0.01, *p ≤ 0.001 (Student’s t-test)

To quantify microbiome-based CRC risk, we developed an MRS incorporating seven key microbial biomarkers. The score was constructed using the Baxter dataset and demonstrated strong discriminatory power in separating CRC from adenoma and control groups. This finding was further validated using the independent Zackular dataset (Fig. 4; Table 3). In both cohorts, CRC samples consistently exhibited significantly elevated MRS values compared to non-CRC groups (*p* < 0.001 in discovery; *p* < 0.05 in validation), highlighting the MRS as a robust, non-invasive tool for early detection.

**Table 3:** Mean and standard deviation of the MRS score across control, adenoma, and CRC groups in discovery and validation cohorts

Study	Control	Adenoma	CRC
**Baxter**	0.22	0.34	0.62
**(discovery)**	(SE=0.025)	(SE=0.029)	(SE=0.053)
**Zackular (validation)**	0.4	0.27	0.56
	(SE=0.10)	(SE=0.069)	(SE=0.10)

SE: standard error

Boldface is used solely to highlight the dataset used and the groups

## Discussion

In this study, we establishes a gut-microbiome–driven machine-learning framework that pairs a RF classifier with a MRS to facilitate early detection and risk stratification of CRC and adenomas. By analyzing stool-derived 16 S rRNA profiles from several public cohorts, we characterized community shifts, identified robust microbial biomarkers, quantified CRC risk, and demonstrated that microbiome signatures can augment existing screening strategies. PCA revealed clear separations—particularly between the CRC group and the control/adenoma groups (Fig. 1A)—suggesting a shift in microbial community structure associated with CRC progression. At the phylum level, Firmicutes, Bacteroidetes, Proteobacteria, Actinobacteria, and Verrucomicrobia predominated across groups (Fig. 1B), with a significantly elevated Firmicutes: Bacteroidetes (F/B) ratio in the CRC group. This increase indicates a shift toward pro-inflammatory dysbiosis and has been correlated with heightened CRC risk. These findings align with previous reports linking an altered F/B ratio to CRC development and support its use as a potential biomarker for early detection [38, 39].

To obtain reproducible biomarkers, we replaced traditional differential-abundance tests—such as linear discriminant analysis effect size (LEfSe) and the Wilcoxon rank-sum test, which neglect the compositional nature of sequencing data [40, 41]—with ANCOM-BC, a bias-corrected approach that adjusts for sampling variability [42], and we supplemented the analysis with chi-square testing. This strategy yielded 109 taxa associated with disease status. Of the seven most discriminative genera, *Porphyromonas*, *Peptostreptococcus*, *Parvimonas*, *Fusobacterium*, and *Collinsella* were enriched in CRC, corroborating their reported tumor-promoting roles [43, 44], whereas *Haemophilus* and *Atopobium* were more abundant in adenomas, suggesting involvement in early neoplastic change. A complementary presence/absence analysis (Fig. 3) revealed CRC-linked microbes that abundance-based methods might overlook, mitigating sparsity and inter-individual variability.

Across the three primary datasets used for model development, the total sample size was 1,181, comprising 454 controls, 373 non-advanced adenomas, 197 advanced adenomas, and 157 CRC cases. RF models trained on these biomarkers achieved strong diagnostic performance, with an AUC of 0.90 in 10-fold cross-validation and 0.82 in external validation (Table 1), while specificity exceeded 0.95 in both settings. The decrease in sensitivity in external validation—particularly for distinguishing adenomas from CRC—likely reflects both the intrinsic challenge of detecting early lesions and population-specific microbiome differences. Clinically, this sensitivity drop highlights the potential risk of false negatives, which could delay intervention in high-risk individuals. Therefore, strategies to mitigate this limitation, such as ensemble modeling, threshold optimization, and integration with complementary screening tools (e.g., FIT), will be essential before clinical deployment. In addition, while the Zackular dataset provided balanced group sizes and detailed demographic and anthropometric data (age, sex, ethnicity, BMI), cancer stage breakdown was not available. The Chinese Yang and Cong datasets were excluded from ML model validation due to the absence of raw sequencing data for standardized processing and lack of adenoma cases, but were suitable for binary MRS validation.

The MRS aggregates diversity metrics—specifically richness, evenness, and community composition—of a disease-associated microbial sub-community identified through rigorous differential abundance analysis. This integrated ecological signature, rather than single-taxon abundance, improves robustness against inter-individual variability. In our analysis, the selected sub-comminity based MRS increased step-wise from controls to adenomas to CRC in the discovery (Baxter) and validation (Zackular) datasets (*p* < 0.001) and was independently confirmed in two Chinese cohorts (Yang, *p* = 3.4 × 10⁻⁶; Cong, *p* = 0.0017; Supplementary Tables 4–5 and Supplementary Fig. 2). This reproducibility across North-American and East-Asian populations underscores the MRS’s portability and highlights its potential utility as a non-invasive, generalizable risk stratification tool. These recent epidemiological findings also emphasize diverse clinical presentations and patient outcomes [10, 11], underscoring the importance of developing non-invasive screening tools adaptable to varied patient populations.

Although the following studies are not microbiome-based, they provide complementary insights that inform the development of integrated early CRC detection and prevention strategies. Recent experimental evidence has identified ribosomal protein L22-like 1 (RPL22L1) as a potential prognostic and therapeutic target in CRC [45]. Knockdown of RPL22L1 in CRC cell lines suppressed proliferation and migration, induced cell-cycle arrest, and promoted apoptosis through inhibition of the mTOR pathway, with in vivo validation demonstrating reduced tumor growth. These mechanistic insights highlight the potential of integrating molecular targets such as RPL22L1 with non-invasive microbiome biomarkers to advance precision screening and intervention strategies. In the therapeutic domain, autocrine motility factor (AMF)-derived peptides—targeting the AMF pathway—have demonstrated potent anti-cancer effects in CRC models, including growth inhibition, ROS induction, and enhanced efficacy when combined with the plant-derived compound glycyrrhetinic acid [46]. This peptide–phytochemical synergy offers a potential therapeutic avenue that could complement microbiome-based early detection, targeting colorectal cancer progression through distinct and complementary molecular mechanisms. From an epidemiological perspective, a recent case–control study in Khyber Pakhtunkhwa, Pakistan, identified high-risk lifestyle and dietary factors—including frequent fast-food consumption, reuse of cooking fats, and tobacco use—as significantly associated with CRC, whereas high vegetable intake, healthy eating habits, and regular sleep patterns were protective [47]. These region-specific risk profiles may contribute to variability in gut microbiome signatures across populations and reinforce the need for geographically tailored prevention strategies alongside universal screening models.

From a clinical implementation and value perspective, our microbiome-based framework is designed to complement—not replace—established screening such as FIT. A practical two-step pathway—FIT for population screening followed by a reflex microbiome assay to triage borderline or FIT-negative results—could improve early detection without materially increasing costs. The same stool sample can be used for both tests, and targeted microbial panels and cloud-automated pipelines can return results within days while minimizing infrastructure, enabling deployment from tertiary centers to community clinics. In population screening, single-sample quantitative FIT typically yields CRC sensitivity ≈ 0.74 [48] and advanced-adenoma sensitivity ≈ 0.23–0.40 at specificity ≈ 0.94–0.96 [6, 49], whereas colonoscopy—though invasive and resource-intensive—remains the reference standard; importantly, pooled sensitivity to detect adenomas ≥ 6 mm is similar between CT colonography with bowel preparation (0.86) and colonoscopy (0.89) [48]. In our study, the microbiome random forest (RF) model achieved AUCs of 0.90 (10-fold cross-validation) and 0.82 (external validation) with specificity > 0.95, comparable to FIT specificity; thus, coupling FIT with microbiome profiling may enhance adenoma detection while maintaining a low false-positive rate. Given its non-invasive nature and ability to capture complementary biological signals, this assay could be applied to average-risk adults in existing screening programs, FIT-negative individuals with persistent clinical suspicion, and higher-risk groups such as those with family history, prior adenomas, or relevant lifestyle factors. Screening intervals could align with current FIT schedules (annual or biennial), with shorter intervals for higher-risk strata; prospective studies are needed to define optimal frequency, refine risk thresholds, and assess cost-effectiveness and adherence across diverse healthcare settings.

Several methodological considerations temper these findings. The datasets targeted different 16 S rRNA regions (V1–V3, V3–V4 or V4), which can influence taxonomic resolution and community profiles due to primer-dependent biases. For example, targeting V1–V3 tends to enrich genera such as *Prevotella*, *Fusobacterium*, and *Streptococcus*, whereas V4–V6 may underrepresent *Fusobacterium* and favor detection of *Campylobacter* and *Enterococcus* [50]. To minimize region‐related batch effects, we (i) reprocessed each dataset from raw reads using a unified QIIME2–EasyMAP pipeline, (ii) trained and validated models only within datasets sharing the same targeted region, and (iii) applied region‐specific taxonomic classifiers based on the SILVA database to harmonize taxonomic calls. We did not directly pool ASV tables across datasets with different target regions to avoid introducing region‐driven artifacts. While these steps reduce primer‐related bias, residual variability from sequencing protocols, sample handling, and cohort‐specific factors may still contribute to performance differences. Future work should standardize sequencing protocols or adopt multi‐region approaches to enhance comparability. Geographic diversity, while enhancing generalisability, introduces dietary and environmental variability that can influence microbial composition; broader sampling will solidify global applicability. Upstream preprocessing differences also persist despite ANCOM-BC correction, underscoring the need for consensus pipelines. Finally, integrating metagenomics, metabolomics and host factors could bolster sensitivity—especially for precancerous lesions—and provide deeper mechanistic insight. Given that adenoma detection sensitivity remains a key limitation, strategies to address this include incorporating functional and strain-level microbial profiles to capture subtler biological differences, integrating multi-omics data (e.g., metagenomics, metabolomics, host transcriptomics) to provide complementary mechanistic insights, and expanding cohort diversity to capture a broader range of adenoma-associated signatures. These enhancements could strengthen model robustness and improve its utility for early-stage lesion detection.

## Conclusions

Bias-aware biomarker discovery combined with RF modelling and an interpretable MRS demonstrates that gut microbiome signatures can reliably differentiate CRC and adenoma from healthy states, stratify individual risk, and complement existing screening strategies. By leveraging stratified cross-validation within discovery datasets to preserve class proportions and avoiding resampling during external validation, our approach maintained high specificity in independent cohorts while revealing sensitivity challenges—particularly in detecting early lesions—under real‐world prevalence conditions. Continued refinement—particularly to improve sensitivity for early lesions—and prospective validation should facilitate clinical adoption, ultimately enhancing personalised surveillance and improving colorectal cancer outcomes.

## Supplementary Information

Supplementary Material 1. 

Supplementary Material 2.

Supplementary Material 3.

Supplementary Material 4.

Supplementary Material 5.

Supplementary Material 6.

Supplementary Material 7.

## Data Availability

We incorporated five publicly available 16 S rRNA datasets spanning North American and Chinese cohorts. Specifically, the Baxter study has its raw reads deposited in the NCBI SRA (accession SRP062005) and includes samples from the USA and Canada [26]. The Dadkhah study likewise deposits raw reads in the NCBI SRA (accession PRJNA534511) and comprises samples from USA [28]. The Zackular study provides its raw FASTQ files in the article’s supplementary materials and also includes participants from the USA and Canada [25]. The Yang dataset offers only an ASV table in its supplement and focuses on a Chinese cohort [29]. Finally, the Cong dataset stores raw reads in NCBI SRA (accession SRP133809) and represents a Chinese populations [27].

## Code availability

The code used in this study is provided in a public repository: 10.5281/zenodo.15638788.
