# Supplementary figures and images for: Gut microbiome-based machine learning model for early colorectal cancer and adenoma screening
Source: Gut Pathog. 2025 Oct 8;17:80. doi: 10.1186/s13099-025-00750-z (PMC12509340; doi:10.1186/s13099-025-00750-z)

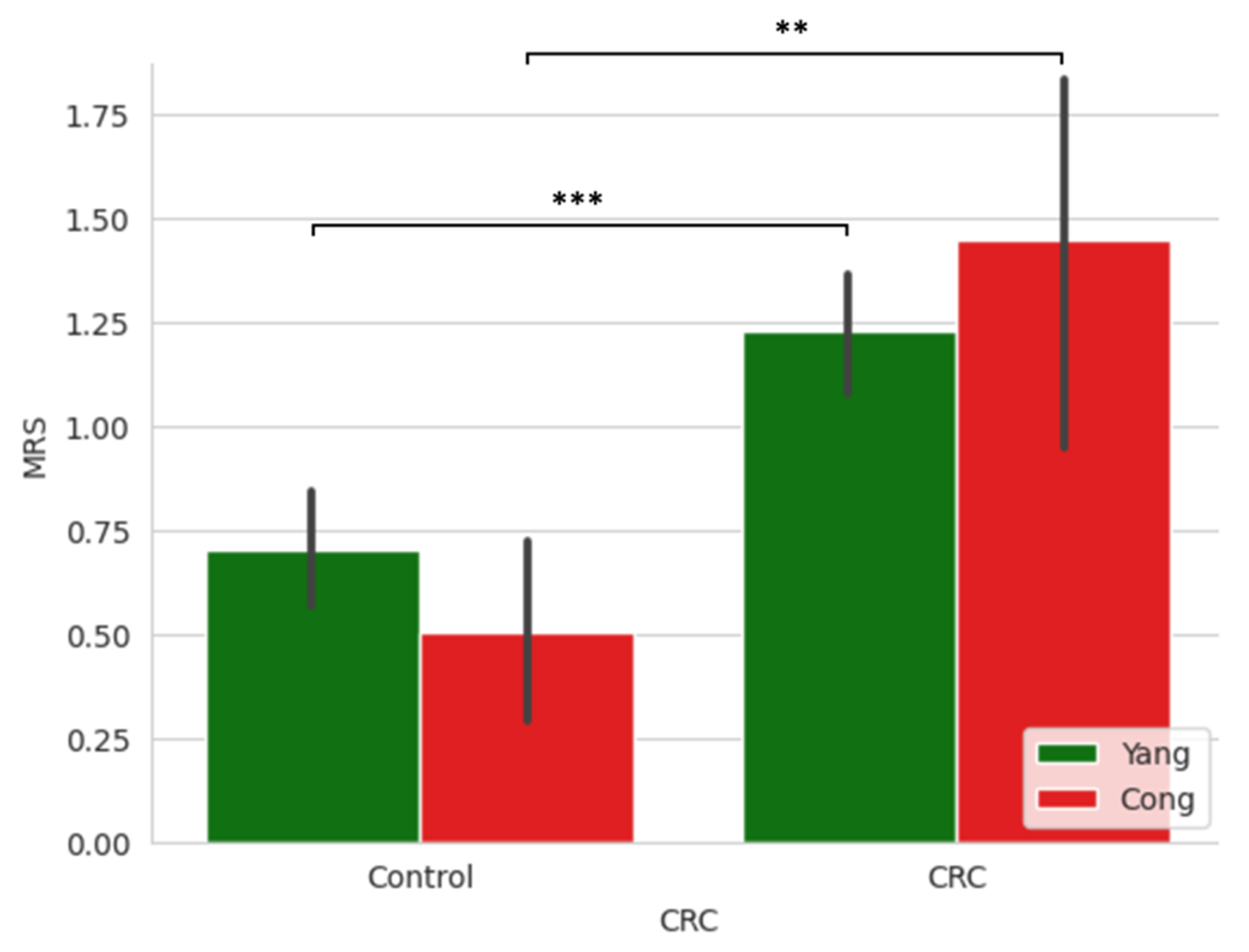

Supplement: Supplementary file 1 — Supplementary Material 1. [file 13099_2025_750_MOESM1_ESM.tif]

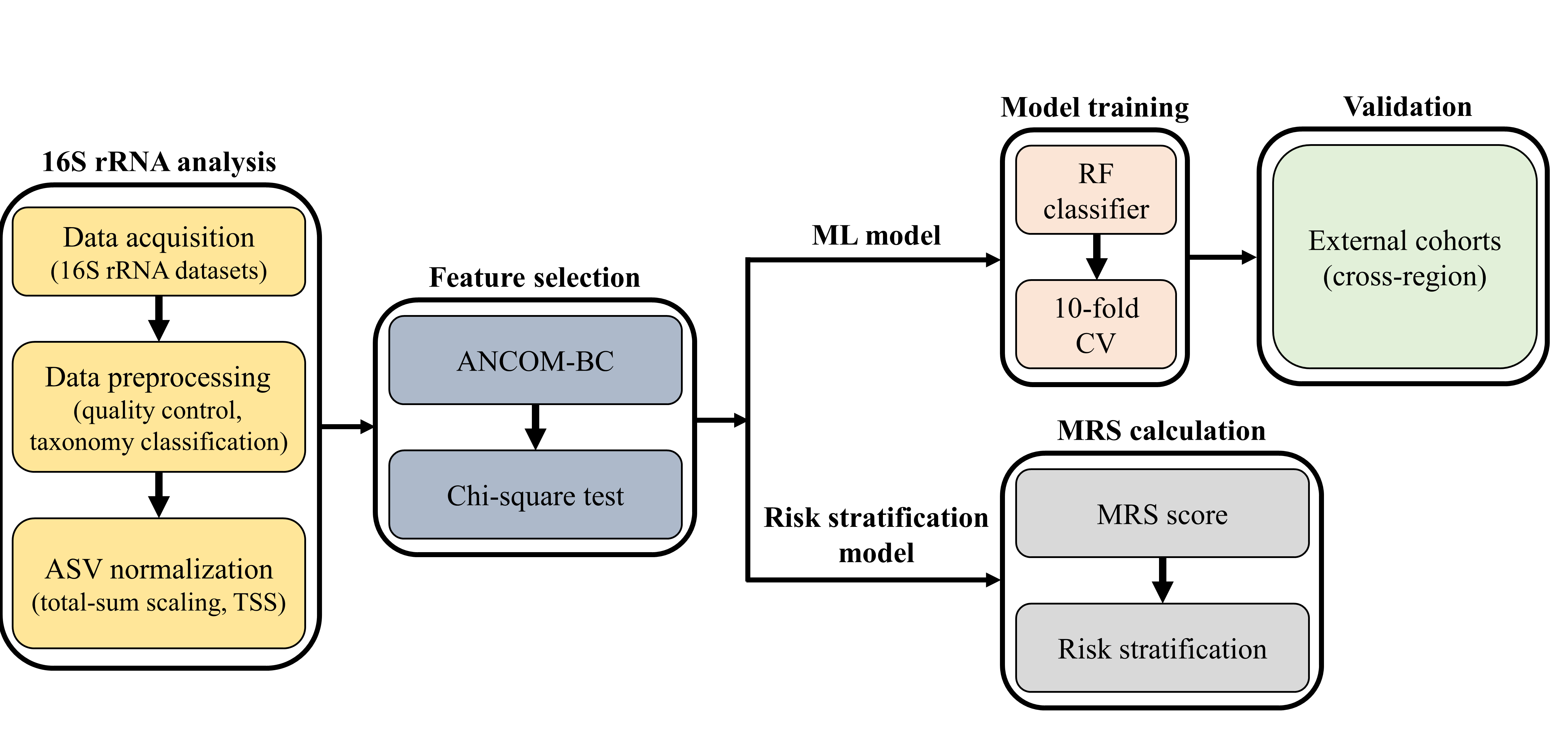

Supplement: Supplementary file 6 — Supplementary Material 6. [file 13099_2025_750_MOESM6_ESM.tif]
